# Cryptochrome expression in avian UV cones: revisiting the role of CRY1 as magnetoreceptor

**DOI:** 10.1038/s41598-021-92056-8

**Published:** 2021-06-16

**Authors:** Atticus Pinzon-Rodriguez, Rachel Muheim

**Affiliations:** Department of Biology, Lund University, Biology Building B, 223 62 Lund, Sweden

**Keywords:** Colour vision, Retina, Sensory processing, Visual system, Circadian regulation

## Abstract

Cryptochromes (CRY) have been proposed as putative magnetoreceptors in vertebrates. Localisation of CRY1 in the UV cones in the retinas of birds suggested that it could be the candidate magnetoreceptor. However, recent findings argue against this possibility. CRY1 is a type II cryptochrome, a subtype of cryptochromes that may not be inherently photosensitive, and it exhibits a clear circadian expression in the retinas of birds. Here, we reassessed the localisation and distribution of CRY1 in the retina of the zebra finch. Zebra finches have a light-dependent magnetic compass based on a radical-pair mechanism, similar to migratory birds. We found that CRY1 colocalised with the UV/V opsin (SWS1) in the outer segments of UV cones, but restricted to the tip of the segments. CRY1 was found in all UV cones across the entire retina, with the highest densities near the fovea. Pre-exposure of birds to different wavelengths of light did not result in any difference in CRY1 detection, suggesting that CRY1 did not undergo any detectable functional changes as result of light activation. Considering that CRY1 is likely not involved in magnetoreception, our findings open the possibility for an involvement in different, yet undetermined functions in the avian UV/V cones.

## Introduction

Cryptochromes (CRY) are flavoproteins well known for their role in the regulation of circadian activity in diverse animals (reviewed by^1–3^). They have also been proposed as the candidate magnetoreceptor proteins underlying the light-dependent magnetic compass of animals^4^. Magnetoreception of the light-dependent magnetic compass is suggested to be based on a radical-pair process which involves a light-induced electron transfer between two reaction partners, and thereby the creation of an excited-state radical pair which the magnetic field can act upon^4–8^. Assuming that such radical-pair-based magnetoreceptors are arranged in an ordered, spherical array, with different receptors aligned at different angles to the magnetic field, the animal would be able to perceive a sensory pattern centrally symmetric to the magnetic field lines^4,9–11^. Such a magnetic modulation pattern would provide information about the spatial alignment, but not the polarity, of the magnetic field lines, which is used by animals with an inclination compass, such as birds, amphibians and insects^10,12–14^. In birds, the eye with its semi-spherical shape and the highly ordered array of cells in the retina has been considered as the most likely location where magnetoreception may take place^4,8,11^.

Cryptochromes are the only known vertebrate photopigments that are able to form spin-correlated radical pairs upon light excitation that last long enough for a magnetic field of the strength of the Earth’s magnetic field to affect the interconversion between the singlet and triplet excited states of the radical pairs^4,15,16^. Several members of the cryptochrome gene family have been found to be expressed in the retinas of both migratory and non-migratory birds^17–24^: the (vertebrate) type II cryptochromes Cry 1, with its two isoforms CRY1a and CRY1b, and CRY2, and the type IV cryptochromes CRY4a and CRY4b. Until recently, CRY1a was considered the most promising candidate magnetoreceptor in birds^25^. Evidence of CRY1a presence in the outer segments of SWS1-opsin (OPNSW1) expressing ultraviolet/violet (UV/V) cones across the retinas of domestic chickens (*Gallus domesticus*) and European robins (*Erithacus rubecula*) agreed with the involvement of CRY1a in radical-pair-based magnetoreception^26–28^. In birds exposed to full-spectrum or monochromatic light with peak wavelengths between 373 nm UV and 590 nm yellow light prior to dissection CRY1a and SWS1 colocalised in the retina, whereas in birds exposed to 645 nm red light or in darkness only SWS1 opsin, but no CRY1a, could be detected^27,28^. These findings at least partially agree with behavioural experiments showing that the light-dependent magnetic compass in birds is only operational under wavelengths between about 370 nm UV and 565 nm green light, but not under lights of longer wavelengths^29–32^. Apart from the presence of CRY1a under 590 nm yellow light, the wavelength-dependent presence of CRY1a is also compatible with the photocycle and formation of different redox states of the cryptochrome cofactor FAD (Flavin Adenine Dinucleotide). Thereby, the fully oxidized state FAD_OX_ is reduced to a semi-reduced state, the neutral semiquinone radical FADH●, by the absorption of light in the UV and blue spectrum (peaks at about 360 nm and 470 nm). The neutral semiquinone radical FADH● in turn is reduced to the fully reduced state FADH- by the absorption of light from the UV to red (peaks at about 495 nm and 580 nm), which is then re-oxidized in darkness to the fully oxidized state FAD_OX_^33–37^. The radical pairs can only be affected by the magnetic field in the semiquinone form, formed either during photoreduction of FAD_OX_ to FADH● or during re-oxidation of FADH- to FAD_OX_^35,38^. In both *Arabidopsis* and *Drosophila* cryptochromes, light activation of FAD has been shown to result in a conformational change of the C-terminal end of the cryptochrome protein^39–43^. Since Niessner and colleagues observed CRY1a labelling only after exposures to UV to yellow light, but not after exposure in darkness or under red light, and their antibody was directed against the C-terminal segment of the cryptochrome, they proposed that their antibody only detected the protein after a light-triggered conformational change^25–28^. It is worth mentioning that during the revision of this manuscript, Bolte *el at*.^44^ confirmed that CRY1a is present in the outer segments of UV cones in European robins, Eurasian blackcaps (*Sylvia atricapilla*) and domestic chickens, using an antibody directed against the same target as Niessner et al.^26^. However, contrary to Niessner et al*.*’s results, the detection of the CRY1a signal did not differ between light- and dark-adapted retinas, arguing against the involvement of a conformational change of CRY1a^44^.

Despite the presented evidence for a role of CRY1 in avian light-dependent magnetoreception, it has been questioned more recently whether vertebrate type II cryptochromes, which include avian CRY1, have the functional properties to act as the primary magnetoreceptors. Type II cryptochromes are widely considered to be integral parts of the negative feedback loop of the circadian clock in vertebrates by inhibiting CLOCK/BMAL1-driven^2,3,45,46^. Furthermore, they are believed to have a very low binding affinity to FAD and to not be intrinsically photosensitive^47,48^. Last, but not least, CRY1a, CRY1b and CRY2 have been shown to exhibit circadian expression patterns in the retinas of several bird species, which does not exclude, but makes a role in avian magnetoreception less likely^20,22,23,49–52^.

In view of these findings, we reassessed the suitability of CRY1 as magnetoreceptor by localising CRY1 proteins in the retinas of zebra finches (*Taeniopygia guttata*, Reichenbach 1862). Zebra finches have a light-dependent magnetic compass based on a radical-pair mechanism, similar to migratory birds^32,53–55^. We recently showed that in the zebra finch retina expression of *Cry1* and *Cry2* genes, unlike *Cry4* genes, exhibits a clear circadian expression profile, suggesting a role in the circadian regulation of physiological processes rather than in magnetoreception^23^. Here, we examined the cellular localisation and distribution of CRY1 protein across the zebra finch retina and tested whether the detection of CRY1 protein was wavelength dependent by examining the abundance of CRY1 after exposure to monochromatic lights.

## Results

### CRY1 antibody

**Figure 1:**
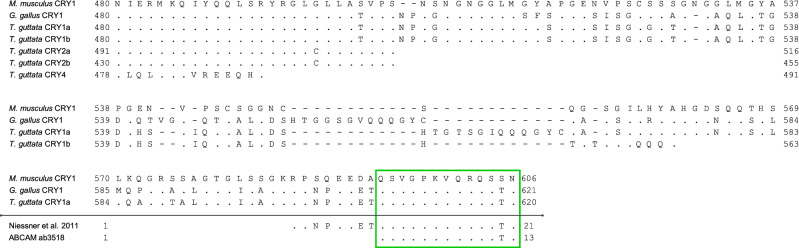
Alignment of the C-terminal amino acids of mouse (*Mus musculus*) CRY1 with chicken (*Gallus gallus*) CRY1 and several zebra finch (*Taeniopygia guttata*) cryptochromes. The green box highlights the target sequence detected by the ABCAM CRY1 antibody used in this paper, as well as the target sequence used by Niessner^26,27,56^, and Bolte^44^. Accession numbers: mouse CRY1 (NP_031797.1), chicken CRY1 (NP_989576.1), zebra finch CRY1a (XP_030118992.2), zebra finch CRY1b (XP_030118993.2), zebra finch CRY2a (XP_030130159.1), zebra finch CRY2b (XP_012429630.1), zebra finch CRY4 (XP_002198533.1).

To detect the presence of CRY1 in the zebra finch retina we used a commercial polyclonal antibody designed to target a peptide unique to CRY1 (Fig. 1). The target sequence is almost identical, albeit shorter, to the sequence used by Niessner^26,27,56^, and more recently by Bolte^44^, to detect CRY1 in retinas of other bird and mammal species. Even though the western blot analysis on total protein extracted from the retinas of the zebra finch with our antibody revealed a single band at a lower molecular weight than the expected size (see supplemental information), the immunofluorescent signal location and pattern coincides with that independently reported by Niessner^26,27,56^, and Bolte^44^ and colleagues, strongly supporting that the antibody used in this paper is likely detecting CRY1.

It is important to note that the alignment shown in Fig. 1 includes different isoforms for CRY1 and CRY2. When we started the current study, no isoforms of CRY1 were known for the zebra finch, which is why we make no differentiation between CRY1 and CRY1a throughout the text. Nevertheless, the alignment clearly shows that the detected epitope corresponds to the CRY1a isoform, as is also the case for the protein detected by Niessner^26,27,56^, and Bolte^44^.

### CRY1 expression in UV cones

**Figure 2:**
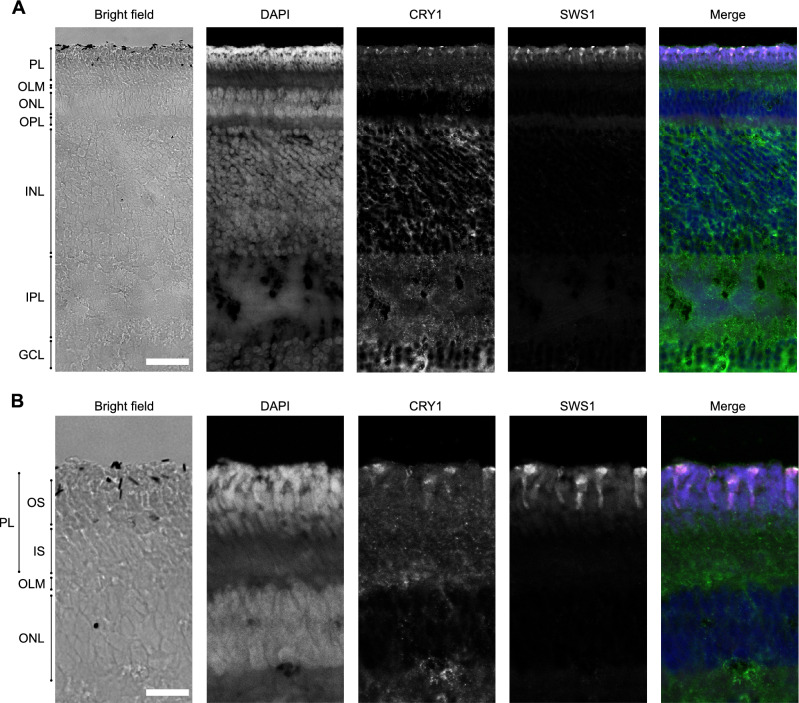
Cross section of the zebra finch retina. (**A**) Overview of the cross section observed with bright field microscopy (first panel) and confocal fluorescence microscopy (second to fifth panel). The fluorescence panels are merged into an artificially coloured merged image (fifth panel) in which blue corresponds to DAPI (including autofluorescence due to collapsed retinal pigment epithelium), green to CRY1 and magenta to SWS1, with colocalisation of CRY1 and SWS1 resulting in white. (**B**) Detail of a cross section imaged as in (**A**). PL (photoreceptor layer), OS (outer segments), IS (inner segments), OLM (outer limiting membrane), ONL (outer nuclear layer), OPL (outer plexiform layer), INL (inner nuclear layer), IPL (inner plexiform layer) and GCL (ganglion cell layer). Bar is 20 µm in (**A**) and 10 µm in (**B**).

Evaluation of cross sections of the zebra finch retina revealed CRY1 immunolabelled cells exclusively in the photoreceptor layer (Fig. 2A, B, third panel). Some non-specific signal in the inner nuclear layer and inner plexiform layer did not seem to be associated with any other retinal cells. We believe it to be background noise present in that channel, or faint autofluorescence since it is also noticeable in the negative control without primary antibody (Fig. S2C). The strong signal visible in the photoreceptor layer in the DAPI channel is most likely due to the collapse of the pigment epithelium layer during dissection and sectioning (also noticeable from the flattened appearance of the outer segments). The retinal pigment epithelium contains lipofuscin, a known source of autofluorescence in the vertebrate retina^57,58^, and perhaps broken oil droplets (Fig. S2C).

**Figure 3:**
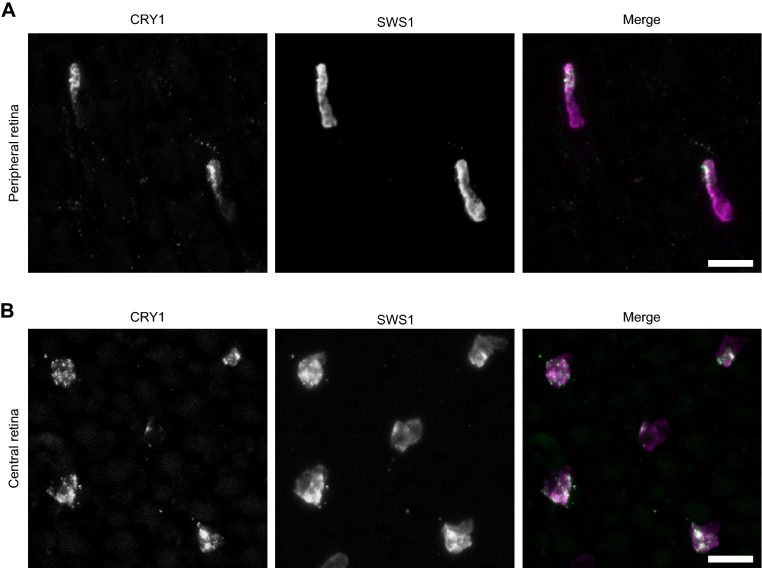
Outer segments of the zebra finch UV cones in a whole mount retina. (**A**) Outer segments of photoreceptors in the peripheral retina. They appear bent and elongated because of the low density of photoreceptors and the absence of pigment epithelium, which, when present, helps to keep them straight. The signal from CRY1 appears only at the tip of the outer segment (first panel), while the UV opsin is visible across the full length of the outer segment (second panel). (**A**) Outer segments of the photoreceptors in the central retina. Here, the photoreceptors are not bent, because their high density keeps them packed and straight despite the absence of pigment epithelium. The merged images are coloured as in Fig. 1. Bar 10 µm.

The CRY1 signal colocalised with the SWS1 immunolabelled cells (Fig. 2A, B, fourth and fifth panel), confirming that CRY1 is present exclusively in UV cones of zebra finches, and in no other photoreceptors. Like SWS1, CRY1 was located in the outer segments of the UV cones, but it was less densely packed than SWS1 and accumulated towards the tip of the outer segment (Fig. 3). This difference in localisation of CRY1 and SWS1 signals in the outer segment is clearly visible in the bent photoreceptors of the flat-mounted, peripheral retina (Fig. 3A). It is not visible in flat mounts of the central retina due to the photoreceptors being straight (Fig. 3B).

**Figure 4:**
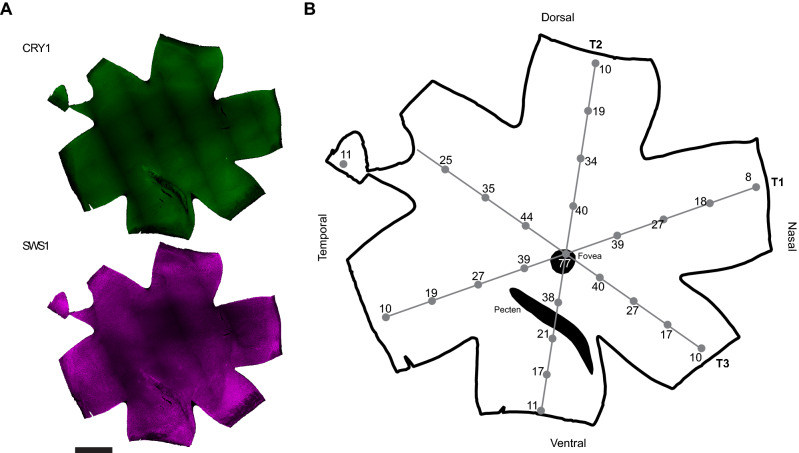
Example of a whole-mounted retina of a zebra finch. (**A**) Whole-mounted retina showing a positive signal for CRY1 (upper image in green) and for SWS1 opsin (lower image in purple). The image is from a left eye retina, mounted with the photoreceptors side up. The banded pattern is an artefact of the digital reconstruction of the entire image from smaller images at higher magnification. The darker regions towards the centre of the retina correspond to thicker remains of pigment epithelium. (**B**) Schematic of the entire retina shown in (**A**). The grey lines and dots show the sampling transects (T1, T2 and T3) and locations where the immunopositive signals were counted. The numbers (× 100) give the counts of positive CRY1/SWS1 cells calculated for a 1 mm^2^ area at the respective locations of the retina. Bar: 2 mm.

**Figure 5:**
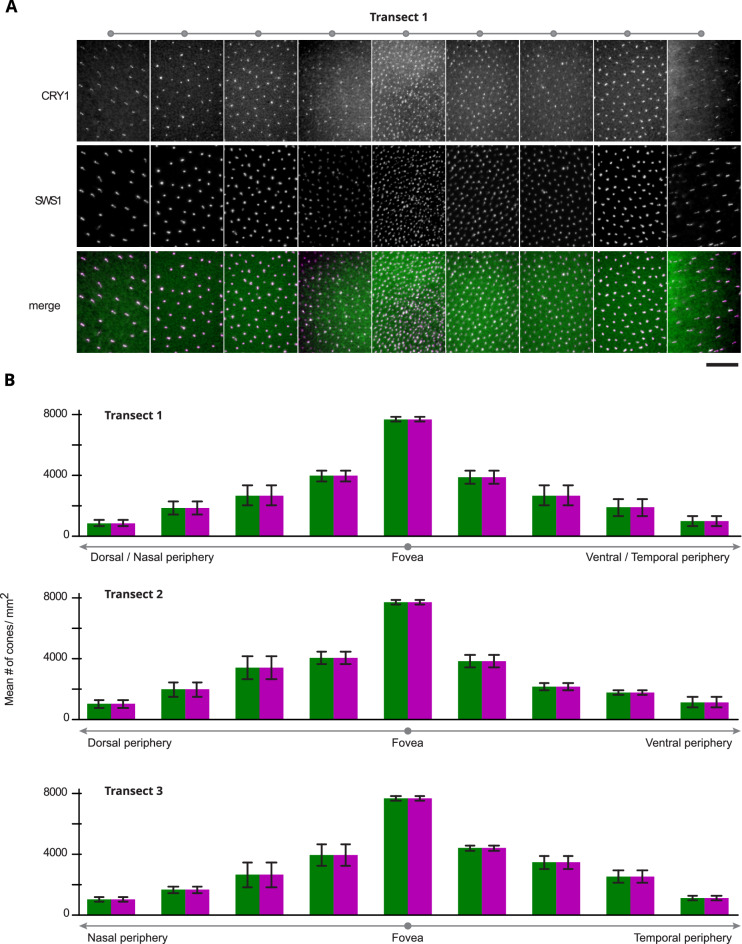
Quantification of the co-localisation of CRY1 and SWS1 in the zebra finch retina. (**A**) Example of sampled areas across Transect 1 of a whole-mounted retina (see Fig. 3B for position of sampling transects and counting locations). The upper row shows CRY1 positive cells, the middle row SWS1 positive cells, and the bottom row the merged images (CRY1 in green, SWS1 in purple and colocalisation is seen as white). (**B**) Mean number (± standard deviations) of immunopositive cells per mm^2^ for each sampling location along the three transects. The counts are based on six retinas from four individual birds (see Table S1). Bar: 30 µm.

The distribution of CRY1-positive cells was identical to the distribution of UV cones across all retinas examined (Fig. 4A). The evaluation of transects to quantify the colocalisation of CRY1 and SWS1 (Fig. 4B) did not reveal any differences in distribution between left or right eyes, or sex of the birds. In all evaluated retinas we found the same pattern of a peak density of the CRY1/SWS1 positive cells in the vicinity of the fovea and a concentric decline towards the periphery of the retina (Fig. 5A). The density of the co-labelled cells was slightly higher in the central, dorsal-temporal area compared to the other areas of the retina at equal distances from the fovea (Fig. 5B).

### Effect of pre-exposure to monochromatic light on CRY1 localisation

**Figure 6:**
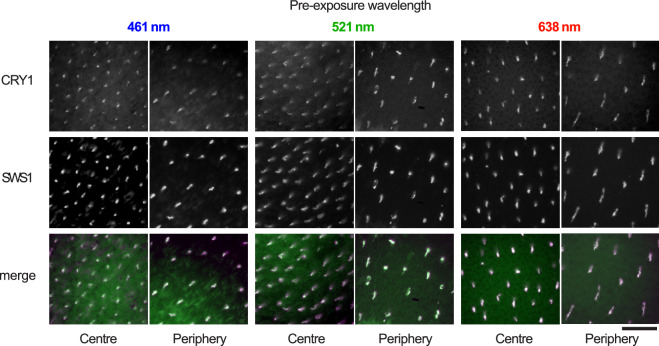
Colocalisation of CRY1 and SWS1 in retinas of zebra finches exposed to monochromatic illumination. The upper row shows CRY1-positive cones, the middle row SWS1-positive cells, and the bottom row shows the merged images (CRY1 in green, SWS1 in purple and colocalisation is seen as white). The panels illustrate examples from birds exposed to 461 nm blue light (left), 521 nm green light (centre) and 638 nm red light (right). For each wavelength spectrum, an example from the central retina and one from the peripheral retina is shown. Bar: 30 µm.

We found no observable differences in the colocalisation of CRY1 and SWS1 opsin, neither in the central fovea nor the periphery in any of the retinas of birds exposed to monochromatic light of 461 nm (blue), 521 nm (green) or 633 nm (red) prior to and during dissection (Fig. 6). CRY1 was detected in all cases irrespective of the wavelength during pre-exposure. There were no differences in CRY1 localisation between left and right eyes, or between male and female individuals.

## Discussion

The aim of this study was to reassess the suitability of CRY1 as the primary magnetoreceptor in the retina of birds. Our objectives were to identify the distribution and the cellular localisation of the CRY1 proteins in the retinas of zebra finches and to test whether the localisation of CRY1 was wavelength dependent. Our findings confirm earlier reports from migratory songbirds and chickens that CRY1 is located in the outer segments of UV cones throughout the avian retina. However, we could not substantiate earlier reports of wavelength-dependent effects on CRY1 localisation.

### CRY1 localisation in outer segments of UV cones

Our observation that CRY1 was present in all SWS1-labelled UV cones in the retinas of all zebra finches examined confirms earlier reports of CRY1a in UV cones of European robins, Eurasian blackcaps and V cones of chickens^26–28,44^. The occurrence of CRY1 proteins in only the UV cones, and no other retinal tissue, in zebra finches strongly suggests that our CRY1 antibody exclusively labelled the C-terminal of the CRY1a isoform of the protein (cf.^25–28^). CRY1b, in contrast, has been found in the retinal ganglion cells, displaced ganglion cells, and inner segments of some unspecified photoreceptors in several species of migratory songbirds and pigeons^59,60^ (see also^61^).

The localisation of CRY1 near the tip of the outer segments of the UV cones is supported by the close proximity to the UV opsins labelled with SWS1. The SWS1 antibody, like opsin antibodies in general, is known to only label the opsins once they are fixed in the membrane of the discs of the outer segment of the cones, but not while being transported from the nucleus in the inner segment to the base of the outer segment, where they are integrated in the membrane of the discs of the outer segment (reviewed by^62^). Our findings only partly agree with the study by Niessner and colleagues^26^, which reported CRY1a detection along the full length of the outer segments of V cones in chickens, and not only at the tip of the outer segments. The same happens with Bolte’s results in blackcaps and robins, where the CRY1 signal also appears in the full length of the outer segment^44^, and not just at the tip of the UV cones as in our results. This difference may originate either from species-specific results, or because the antibodies, despite being designed against a similar region, are not identical. The antiserum used by Niessner^26^ and Bolte^44^ was made against a 20 aa peptide in the C-terminal region of the CRY1a sequence, while the antibody we used in this study was a commercial polyclonal anti-CRY1 designed against 12 aa peptide, in the same region of the mouse CRY1 sequence and fully homologous to their 20 aa peptide (Fig. 1). This leaves an 8 aa peptide that is recognized by Niessner’s and Bolte’s antibodies but not ours. Regardless of such differences, the localisation of the CRY1 protein far from the nucleus in the inner segment argues for a function not directly involved in the negative feedback loop of the circadian clock, one of which could be magnetoreception.

According to the original radical-pair model, the putative magnetoreceptors should ideally be fixed along at least one molecular axis and be evenly distributed across a hemisphere, like the avian retina, to allow for the comparison of reaction yields of radical pairs with different alignments relative to the magnetic field^4,8,11^ (note, however, that some of these requirements are not necessarily needed^55,63–65^). These requirements are met by the localisation of CRY1 in the UV cones of the zebra finches, assuming that the CRY1 proteins are aligned roughly along the same axis in the cone outer segments. UV/V cones are in theory ideal candidate locations for cryptochrome-based magnetoreceptors, as pointed out earlier^26,66^. Their transparent oil droplets do not filter out UV light^67,68^, thus light in the UV and blue spectrum can reach the FAD in the cryptochromes, which show a high absorption of light in this wavelength range (reviewed by^2,37,38^). Also, UV/V cones are the least abundant photoreceptors in the avian retina (max. 10%, depending on species; e.g.,^68–70^), which would minimize interference of light-dependent magnetoreception with vision (but see below).

### Distribution of CRY1 across retina

We found an up to nine times higher density of CRY1/SWS1 cones in the fovea of the zebra finch retina compared to the periphery, which agrees well with the general density distributions of cones in retinas of passerine birds, which usually peak in one or two foveas and decrease towards the periphery of the retina^71–74^. The fovea is an important area in the visual field with a high density of cones and none or few rods^75^. This area of improved visual acuity is used for various visual tasks that require high-resolution vision, like food detection or obstacle avoidance^76^. The higher density of CRY1-positive UV cones in the fovea of the zebra finch retina would suggest that, if they were magnetoreceptors, magnetic compass information to be perceived at a higher spatial resolution when viewed through the fovea. This may improve the detection of the magnetic field but could also pose a possible caveat in that the magnetic modulation pattern could interfere with important visual tasks. This will largely depend on how the signals from the CRY1 and UV receptors are processed [see^66^, and discussion on the localisation of CRY1 below].

### Effect of monochromatic light on CRY1 localisation

One of the key indications for an involvement of CRY1 in avian magnetoreception was based on the observation that CRY1 could only be detected in chicken retinas after exposure to UV to yellow light, but not after exposure to red light or darkness^27,28^. Exposure of zebra finches to 461 nm (blue), 521 nm (green) or 633 nm (red) light for one hour prior to dissection did not result in any differences in immunodetection. We detected the CRY1 protein in the retinas of zebra finches exposed to any of the light conditions, irrespective of the spectrum and the location in the retina (centre or periphery; Fig. 5). This came as a surprise, since our primary antibody was designed to detect the same region (C-terminal of CRY1), as the one used by Niessner and colleagues^27,28^. In both cases, the same antibody against SWS1 was used, in similar concentrations. Niessner and colleagues argued that their CRY1a antibody detected the CRY1a protein only after it had undergone a conformational change in the C-terminal upon activation by UV to yellow light, since they detected CRY1a only after exposing the birds to UV to yellow light prior to dissection, but not after exposure to darkness or red light^27,28^. Nevertheless, Bolte and colleagues^44^ found the same discrepancy when testing their antibody on light and dark adapted retinas. No difference was noted after the different light treatments, which made them conclude that there was no evidence of the antibody detecting only the light-activated form of CRY1a.

The assumption that CRY1 undergoes a conformational change was based on evidence from plant (*Arabidopsis*) and type I (*Drosophila*) cryptochromes, which are both known to be directly light sensitive^41–43,77^. We are not aware of any studies showing that vertebrate type II cryptochromes undergo a conformational change in the C-terminal as a result of light activation. On the contrary, they are suggested to be vestigial flavoproteins which do not stably bind FAD and use the C-terminal for interactions with other clock proteins instead^45–47,78^. Assuming that the CRY1 in the outer segments of the UV/V cones are located too far away from the nucleus to be directly involved in the circadian clock, the C-terminal should be detectable to antibodies under any light condition. We observed that the signal from the immunolabelled cells in some regions of our whole mounts was masked by the presence of remnants of the pigment epithelium. Despite being removed to the most extent and bleached to avoid darkening of the preparation, the pigment epithelium interfered with the fluorescent signal of the marked cells, making it almost not differentiable from the background, and therefore easy to be misinterpreted as non-labelled tissue. Our own immunostainings of retinas of robins and chickens will have to show whether this may be the explanation for the discrepancies between studies, or whether differences between the antibodies or bird species are responsible.

### Localisation of CRY1 in the avian UV/V cones suggests unique function

Based on the cellular localisation of the CRY1 proteins at the tip of the outer segments of the UV cones, CRY1 could well be the thought-after candidate magnetoreceptor of the light-dependent magnetic compass^25^. However, the high density of the CRY1-containing UV cones in the central retina is not necessarily in support of a role in magnetoreception, even though it does not exclude this possibility. The molecular and functional properties of CRY1 also argue against its involvement in light-dependent, radical-pair-based magnetoreception. Nevertheless, the possibility remains that CRY1 might be involved in signal transduction further downstream in the signalling cascade.

If CRY1 is not involved in light-dependent magnetoreception in birds, why is it present in the outer segment of all UV cones across the entire retina of the zebra finch, but in none of the other photoreceptors or retinal cells? Together with the reports of CRY1a existance in UV cones of European robins and V cones of chickens^26–28^ our findings suggest that CRY1 likely has a very specific function which is unique to cones expressing the SWS1 pigment and which is not required in any of the other cone or rod photoreceptors, unless other cryptochromes are expressed in those photoreceptors instead. However, to date there is no convincing evidence that either CRY1b or CRY2 are expressed in avian photoreceptors. CRY1b has been reported in ganglion cells and a few displaced ganglion cells in retinas of pigeons (*Columba livia*), European robins and Northern wheatears (*Oenanthe oenanthe*)^59,60^, and possibly in the inner segment of photoreceptors, but this latter observation was only made by one of the groups^59^ and could not be substantiated by the other group^60^. Thus, all evidence points towards a very specific function of CRY1 in only UV/V cones. Interestingly, CRY1 has also been found in short-wavelength sensitive SWS1 (S1) cones in representatives of some groups of mammals (Canidae, Mustelidae and Ursidae within Carnivora, and Hominidae, some Cercopithecidae, and possibly Lemuridae and Callitrichidae within Primata)^56^. It might suggest that the expression of CRY1 is a more widespread feature of SWS1-expressing cones, common to birds and mammals, and possibly vertebrates in general.

SWS1-expressing photoreceptors are unique in that they absorb light in the UV to V spectrum^79–81^, which is the visible light spectrum with the highest energy and has been shown to damage the retina of vertebrates^82^. The vertebrate ancestral state of SWS1 opsins is suggested to be UV sensitive, but was independently replaced by V sensitivity in various lineages (reviewed by^79,83^). These include birds which likely possessed an ancestral V-sensitive pigment with certain lineages secondarily regaining UV sensitivity^83^. Since both the UV and V cones of birds contain transparent oil droplets which contain no carotenoids and do not filter short-wavelength light^72^, CRY1 located in these cones could possibly be involved in UV/V-light protection. However, in mammals UV-light below 400 nm is often absorbed by the cornea and lens^84^, and there does not appear to be any relationship between CRY1 expression and the degree of UV light below 400 nm reaching the SWS1 cones in different groups of mammal^60^.

Other possible functions of the CRY1 proteins in the outer segments of UV/V cones in birds could be linked to the growing body of evidence that cryptochrome proteins, independent of their role as circadian clock regulators, are directly involved in various metabolic processes, like e.g., glucocorticoid signalling^85^, modulation of the cAMP pathway^86^, and DNA damage response (reviewed by^87^).

## Conclusions

Based on the overall detection of CRY1 in the UV cones of the entire retina of zebra finches, CRY1 fulfils the requirements of the radical-pair model for a spatial distribution in a hemisphere, thus could be a candidate magnetoreceptor of the light-dependent magnetic compass in birds based on this criterion alone. However, considering that CRY1 is expressed in a clear circadian rhythm and might not be able to harvest light on its own, it is unlikely the primary magnetoreceptor initiating the radical-pair mechanism, even though the possibility remains that CRY1 might be involved in signal transduction further downstream in the signalling cascade. Nevertheless, the unique localisation of CRY1 at the tip of the UV cones in zebra finches suggests an exclusive function, which however remains to be determined.

## Materials and methods

The experimental procedures carried out in this work were planned and performed in accordance with the ARRIVE guidelines, where applicable.

The birds were handled and terminated for tissue extraction following Swedish ethical guidelines approved by the Malmö-Lund Animal Ethics Committee (permits M 24–16, and M 108–16).

### Experimental animals and light exposure conditions

The zebra finches belonged to a permanent captive breeding colony at Stensoffa Field Station, near Lund (Sweden). The birds were kept indoors under full-spectrum, indoor light conditions under a constant 12 h:12 h light–dark cycle for at least 7 days prior to dissection. The birds belonged to a heterogeneous group of sexually mature individuals (> 6 months old, 12 males and seven females) (Table S1. All retina samples were collected during the summer and autumn of 2017 and 2018 11:00 and 12:00 CET. 4 extra males were dissected for antibody validation controls and Western Blots.

Control birds (seven males and three females) were taken directly from the holding cages for tissue collection. Birds selected for the monochromatic light experiments (five males and four females distributed among the different monochromatic light conditions—Table S1) were exposed in individual cages to one of the three wavelength spectra for 1 h prior to tissue collection. The dissections were done under the same light spectra as they were treated (full spectrum white light or monochromatic light). We used the same monochromatic light conditions as in our previous study on light-dependent magnetic compass orientation in zebra finches^32^: monochromatic light with peak wavelengths at 463 nm blue, 521 nm green or 638 nm red produced by a LED array (OF-BLR5060RGB300, OPTOFLASH, Łódź, Poland) with a total light irradiance of 15–18 × 10^16^ quanta s^−2^ m^−2^.

### Tissue collection & whole mount preparation

Birds were sacrificed by cervical dislocation followed by decapitation and enucleation of both eyes. Retina dissection, fixation and whole mount preparation followed the method described in^88,89^. Briefly, the eyes were enucleated and immediately submerged in phosphate-buffered saline (PBS) where they remained for the duration of the dissection until fixation. To access the eyecup, a circular cut was made around the ora serrata to remove the cornea and lens. The exposed eyecups were then fixed by immersion in 4% paraformaldehyde in 0.1 M phosphate buffer (PB), pH 7.4, for 2 h at room temperature. The fixed retinas were then placed in PBS, and the sclera was carefully detached and removed from the retina. To flatten the retina, six cuts were made from the periphery of the eyecup towards the centre, to alleviate the curvature. Once flat, the pecten was cut away and the pigment epithelium was mechanically removed as much as possible. Since in most samples a portion of the pigment epithelium was strongly attached to the retina, we bleached the dissected retinas in a solution of 3% H_2_O_2_ in 0.1 M PB overnight in the dark and at room temperature. The bleached retinas were then rinsed and stored in 0.1 M PB until use. A shorter fixation (20 min) was used in a few samples (see Table S1), where the bleaching step was replaced by a dissection in PBS at 37 °C. The aim was to detach the pigment epithelium without the need for the bleaching step, exposing the tissue to a less harsh protocol.

### Cryosectioning

The samples were cryosectioned following standard methods^90^: fixed eyecups were cryoprotected by immersion in a 25% sucrose solution overnight at 4 °C. After this, the eyecups were changed from the sucrose solution and immersed in freezing medium (Neg-50, Richard-Allan Scientific, Thermo Fisher, Hvidovre, Denmark) at room temperature for 10 min to fully coat the tissue. The tissue was further embedded in freezing medium and frozen at − 60 °C. Semi-thin Sects. (10 µm) were cut sequentially in a Microm HM 560 cryostat (Microm, Walldorf, Germany) along the dorsal–ventral axis. The resulting sections were thawed/mounted in chrome alum gelatine-coated microscope slides, dried overnight at room temperature, and stored at − 20 °C until further use.

### Antibodies and immunostaining

To identify which cells expressed CRY1, we used a rabbit polyclonal antibody against the C-terminal end of the mouse CRY1 sequence (ab3518. ABCAM, Cambridge, UK; concentration 1:100). Since the corresponding blocking CRY1 peptide from ABCAM was no longer available, we synthesized a custom peptide spanning amino acids 594–606 of the Mouse Cryptochrome I (QSVGPKVQRQSSN; Capra Science, Ängelholm, Sweden) for blocking controls and antibody validation. Specificity of the CRY1 antibody was verified by Western Blot analysis (see supplementary information). To detect UV-cones in the zebra finch retina, we used a goat polyclonal antibody against the N-terminus of the blue opsin in humans (OPN1SW; sc-14363. Santa Cruz, Dallas, TX, USA; concentration 1:250), which labels the SWS1 opsin in the UV and V cones in birds. Zebra finches have UV cones, not V cones^91^, so we are certain that the signal of the SWS1 antibody labelled only UV cones. Primary antibodies were diluted in 1% Triton X-100, 0.1 M PB solution.

Cryosections were incubated with the primary antibodies overnight at room temperature in a humid chamber. After rinsing the slides in PBS + TritonX100, the sections were incubated with the corresponding secondary fluorescent antibody [anti-rabbit Alexa 647 (1:1000) to label CRY1; anti-goat Alexa 555 (1:1000) to label SWS1 opsin; ThermoFisher] for 1 h in darkness at room temperature. The sections were rinsed again in PBS + TritonX100, followed by PBS and then mounted with Fluoromount-G containing DAPI (SouthernBiotech, Birmingham, USA). The secondary antibody spectrum was selected to avoid autofluorescence artefacts raising from the pigment epithelium (see “Results”).

The whole mounted retinas were incubated with the primary antibodies overnight at room temperature under constant gentle rocking. After rinsing the retinas in 0.1 M PB, the tissue was incubated with the corresponding secondary fluorescent antibody [anti-rabbit Alexa 488 (1:1000) to label CRY1; anti-goat Alexa 555 (1:1000) to label SWS1 opsin; ThermoFisher] for 2 h in darkness at room temperature and with constant gentle rocking. The tissue was then rinsed again in 0.1 M PB and placed on gelatinized glass slides, with the photoreceptor side up, and mounted with Fluoromount-G (SouthernBiotech). Control retinas were incubated in the absence of primary antibody or with CRY1 pre-mixed with its corresponding blocking peptide to assess specificity (Figure S2).

### Image acquisition and analysis

To evaluate the presence and intracellular localisation of the CRY1 proteins, we analysed both cross sections and whole mounted retinas with a Leica SP8 DLS confocal microscope (63 × /1.4 objective) with LAS-X software (Leica, Wetzlar, Germany). In addition to the immunolabelled retinal cross-sections, we acquired images of the periphery and the centre of the whole mounted retina from z-stacks spanning 9 µm, starting from the base towards the tip of the outer segments of the UV cones, up to the point where the signal disappeared.

To assess the distribution of CRY1-positive cells and cones expressing SWS1 opsin throughout the retina, mounted slides were inspected on an AXIOPHOT Fluorescence Microscope (25 × /0.8 and 40 × /1.3 Plan-Neofluar objectives; Zeiss, Oberkochen, Germany). The images were captured with a NIKON DS-fi1c CCD camera with NIS-elements software (Nikon. Tokyo, Japan). Composed images of entire retinas were reconstructed using Adobe Photoshop CS6 (San Jose, CA, USA). We established three transects with seven sampling points each to sample the photoreceptor density across the control retinas and to evaluate the degree of co-expression of CRY1 and SWS1. Each transect covered the retina from one periphery, passing through the central fovea all the way to the opposite periphery, so that they all intersected each other at the fovea. At each sampling point, images of each secondary antibody fluorescence were taken, and the number of positive signals was quantified using a custom particle counting software written in Matlab R2016b (The MathWorks Inc., Natick, MA, USA). The program counted positive cells on a selected region of interest of 0.1 mm^2^. Counts from equivalent transect points from all retinas examined were averaged and presented as a histogram, with standard deviation as error bars.

## Supplementary Information

Supplementary Information.

## Data Availability

The data generated and analysed in the present study are included in the manuscript and its supplementary information files, or available on request from the corresponding author.
