# Supplementary material for: Cryptochrome expression in avian UV cones: revisiting the role of CRY1 as magnetoreceptor
Source: Sci Rep. 2021 Jun 16;11:12683. doi: 10.1038/s41598-021-92056-8 (PMC8209128; doi:10.1038/s41598-021-92056-8)
Supplement: Supplementary file 1 — Supplementary Information. [file 41598_2021_92056_MOESM1_ESM.docx]

Cryptochrome expression in avian UV cones: revisiting the role of CRY1 as magnetoreceptor

# Atticus Pinzon-Rodriguez & Rachel Muheim

### Department of Biology, Lund University, Biology Building B, 223 62 Lund, Sweden.

Biology Building B, 223 62 Lund, Sweden.

Correspondence: atticus.pinzon_rodriguez@biol.lu.se

# Supplement

## Western blot

To validate the CRY1 ABCAM antibody, a western blot analysis was performed to assess specificity of the antibody on the target tissue. Total protein from the zebra finch retina was extracted in RIPA buffer (Thermo Fisher, Hvidovre, Denmark), supplemented with cOmplete EDTA-free protease inhibitor cocktail (Roche, Mannheim, Germany), and homogenized by mechanical means with plastic mortar and pestle for 5-10 min, on ice. The lysate was kept on ice for 1 hour and then centrifuged at 10000 G for 10 min. The whole lysate supernatant was used immediately or stored at -20°C. The protein extract was mixed with 5x Laemmli sample buffer (BioRad, Hercules, USA), and loaded in 10% Mini-PROTEAN TGX polyacrylamide precast gels (BioRad), along with molecular weight markers (Precision Plus Protein Dual Color Standards, BioRad). Gels were run at 100 V for 45 min and then transferred for 7 min into a Trans-blot Turbo midi PVDF membrane, using the Trans-blot Turbo transfer system (BioRad). The membrane was blocked in 5% milk in PBS and then incubated with primary antibody overnight at 4°C. The primary antibody (CRY1 ABCAM) was used at a concentration of 1:1000 alone, or preincubated with the blocking peptide at 3:1 ratio peptide-to-antibody to effectively block it. After incubation, the membranes were washed with PBST and incubated with the secondary antibody (goat anti-rabbit IgG-HRP: sc-2004, SantaCruz, Dallas, TX, USA; concentration 1:5000), for 1 hour at room temperature. The membranes were then incubated with Clarity Max Western ECL Substrate (BioRad) for 1 minute and imaged in a ChemiDoc imaging system (BioRadA). Images were analysed with ImageLab software (BioRad).

We did not have access to an isolated CRY1 protein as positive control for the system. Instead, we used our avian CRY4 expressed protein and avian CRY4 custom made antibody (unpublished data) as a control for specificity and to verify that the CRY1ABCAM antibody was detecting the expected protein, since they both have very similar molecular weights. We compared the total whole retinal lysate with the expressed CRY4 protein by incubating those 2 samples with different antibody combinations: CRY1 ABCAM alone, CRY4cmA alone, CRY1 ABCAM + CRY1peptide or CRY1 ABCAM + CRY4peptide. These combinations allowed us to confirm that each antibody was detecting a specific protein and that there was no cross-detection between similar proteins (Figure S1 and S1.1).

The western blot results confirm that CRY1ABCAM detects a single target corresponding to the CRY1 epitope. The cross-testing with CRY4 custom antibody and cross-blocking with the CRY4 peptide confirms that there is no cross-detection between the two antibodies. The general appearance and location of the immunofluorescence signal in retinal tissue suggests that the antibody is indeed detecting CRY1 (as compared to work from other groups that produced antibodies using a very similar target sequence ^1,2^). Therefore, we are confident that the detected band corresponds to CRY1 and that the discrepancy between the band detected in the CRY4-expressing protein sample (crude extract of overexpressed protein) and the band detected in the whole retinal lysate may be an indication of proteolysis or, less likely, changes in the structure due to posttranslational modifications or the effect of detergents that altered the overall charge of the protein, making it migrate further than it would, based only on its molecular weight ^1,2^. This is supported by the observation that both the CRY1 and CRY4 proteins are detected in the whole lysate at about the same location, as is expected for two proteins that have similar molecular weights (69 kDa for CRY1 and 61 kDa for CRY4).


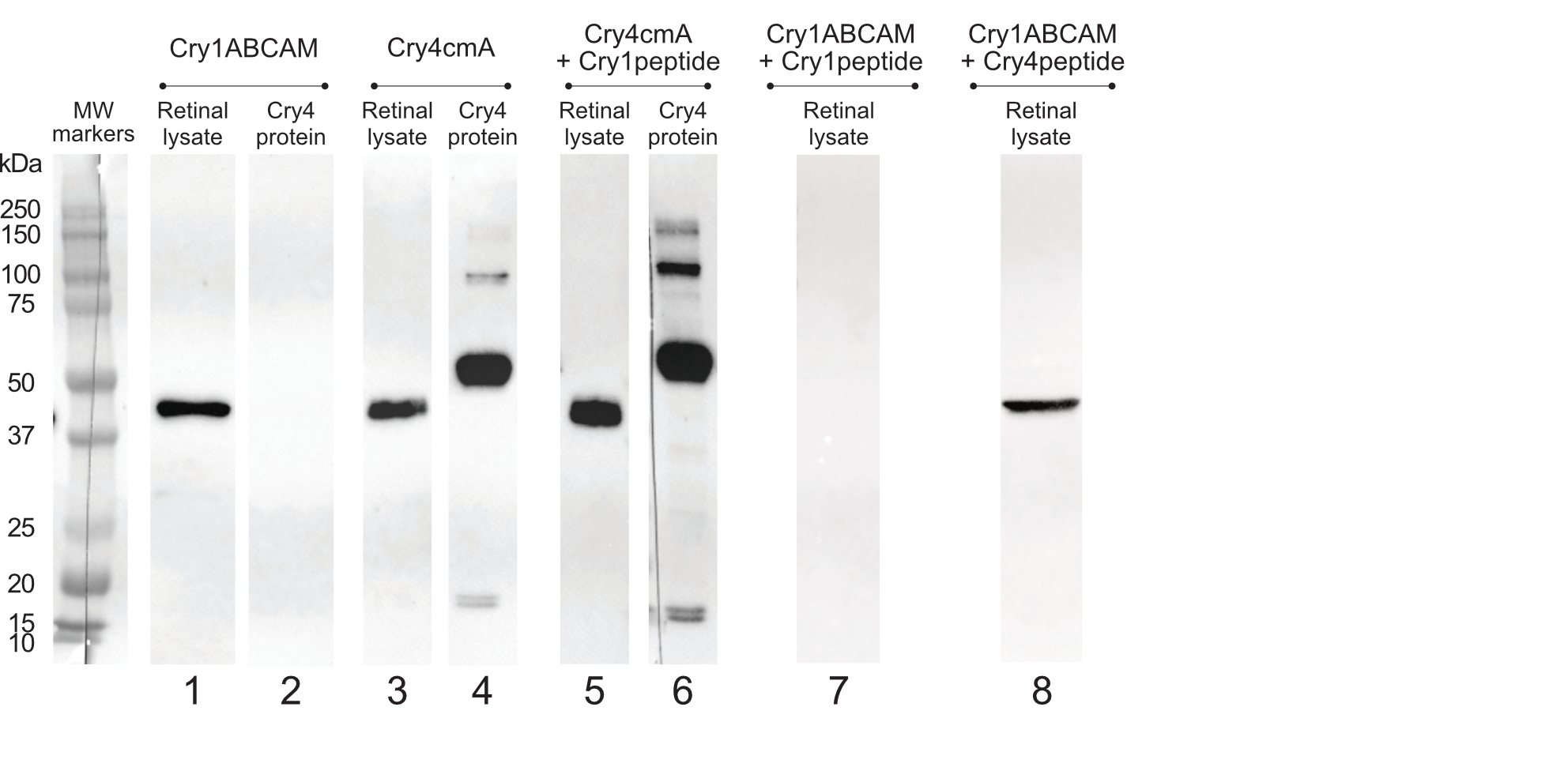


Figure S1. Western blot of whole retinal lysate versus CRY4 expressed protein under incubation with different antibodies. CRY1ABCAM detects a single band in the retinal lysate (lane 1) but does not react to expressed CRY4 protein (lane 2). The CRY4 custom-made antibody (CRY4cmA) detects a single band in the retinal lysate, similar to CRY1ABCAM (lane 3) and also reacts to the expressed CRY4 protein (lane 4) as expected. Blocking the CRY4 antibody with the CRY1 peptide has no effect on the detection of CRY4, either in the retinal lysate (lane 5) or in the expressed CRY4 protein. Incubating CRY1 ABCAM with the CRY1 peptide completely blocks its function and no signal is visible in the retinal lysate (lane 6). Incubating CRY1 ABCAM with the CRY4 peptide shows no effect on the detection of CRY1 in the whole retinal lysate. Source images for each lane presented here are included in Fig S1.1.


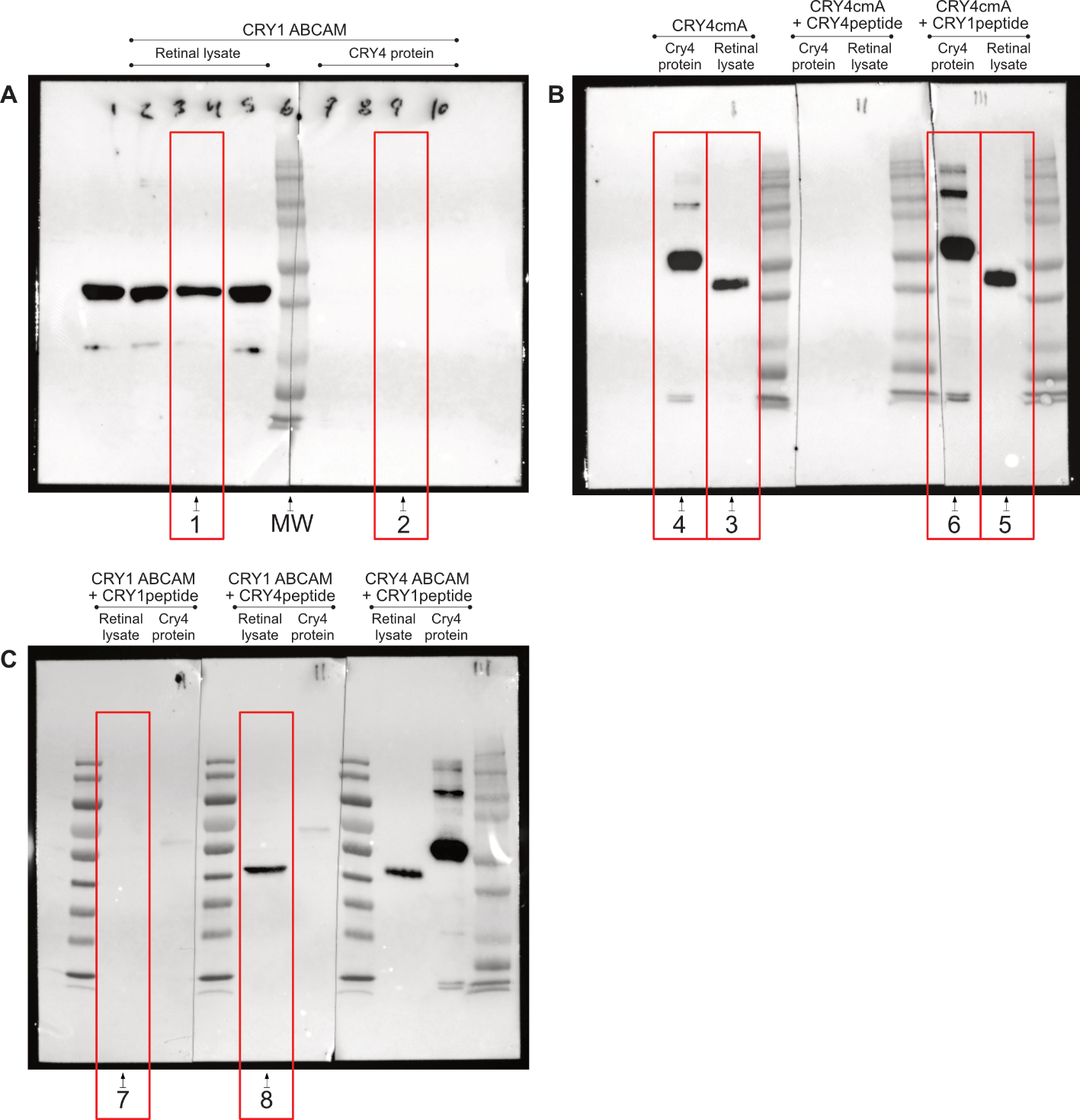


Figure S1.1. Original source images for Figure S1. All the red boxes and numbers with arrows on the lower side of each panel indicate the lanes used to build Figure S1. (A) Test of CRY1ABCAM and different protein extraction buffers. Lanes hand labelled 1-5 contained the full retinal lysate, lanes 7-10 contained CRY4 expressed protein. Lane 6 contained molecular weight markers. (B) Test of CRY4cmA. Section I (hand labelled) shows CRY4 expressed protein, full retinal lysate reacting with the CRY4cmA antibody. Section II (hand labelled) shows no signal either on CRY4 expressed protein nor full retinal lysate lanes, because of the CRY4cmA antibody was pre-blocked with the antigen peptide. Section III (hand labelled) shows CRY4 expressed protein, full retinal lysate reacting with the CRY4cmA antibody, after attempting to block with the CRY1 ABCAM antigen peptide. (C) Test of CRY4cmA and CRY1 ABCAM. Section I (hand labelled) contains CRY4 expressed protein and full retinal lysate, but no signal appears from CRY1ABCAM since it is blocked by the CRY1 ABCAM antigen. Section II (hand labelled) shows signal only for the full lysate when detected with CRY1ABCAM, but no signal arises from the interaction of the antibody with the expressed CRY4 protein. Section III (hand labelled) shows a strong signal from both the full lysate and the CRY4 expressed protein when detected with the CRY4cmA antibody blocked with the CRY1 ABCAM antigen peptide.

## Immunofluorescence controls


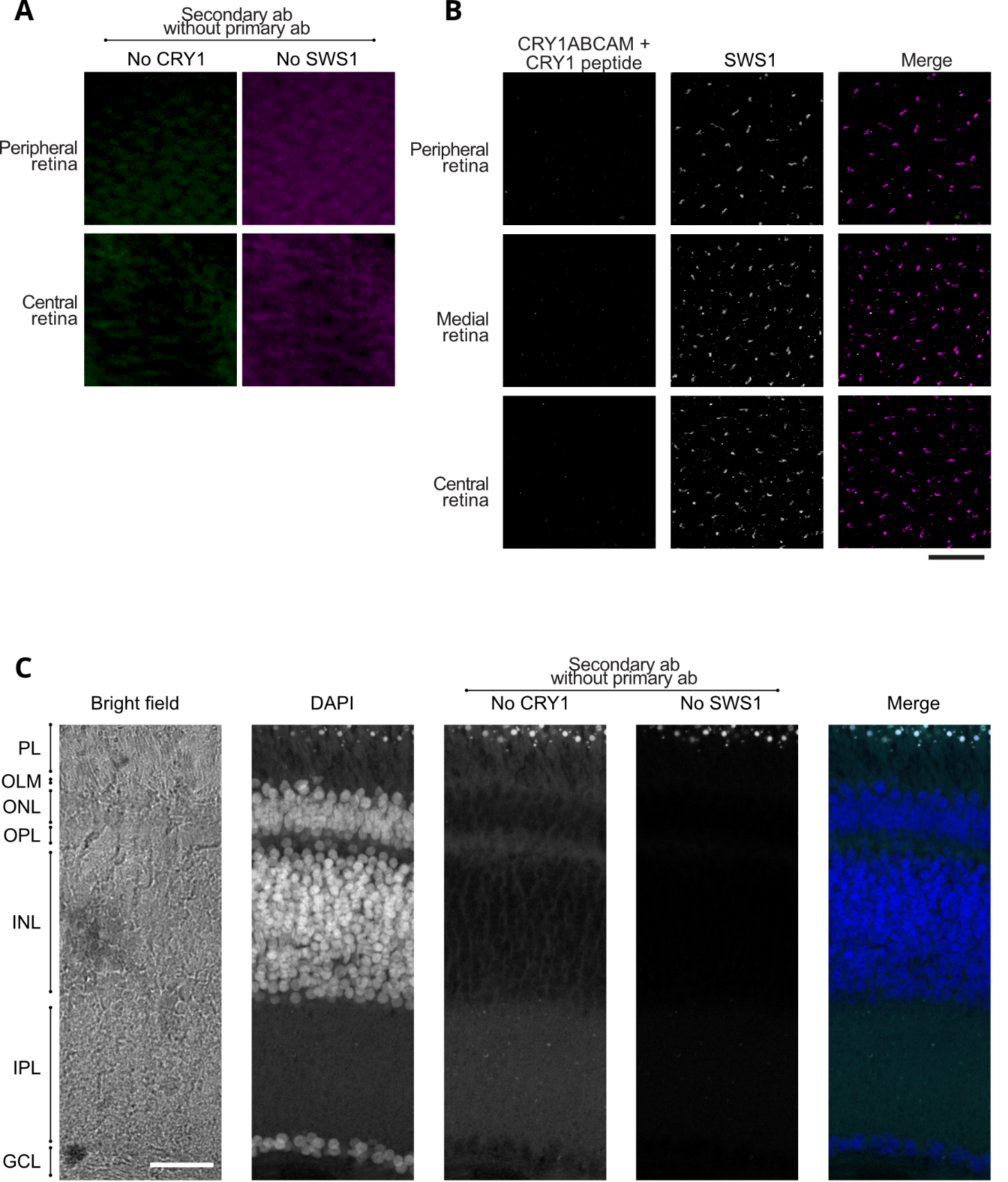


Figure S2. Immunofluorescence controls for the CRY1 ABCAM antibody. (A) Control for unspecific binding of the secondary antibody Alexa Fluor 488 and Alexa Fluor 555 to the tissue without treatment with primary antibody (PBS instead). The absence of signal confirms that the signal comes only from secondary antibodies bound to their corresponding primary species. (B) Control for specific binding of CRY1 ABCAM to its epitope by pre-incubating the antibody with the blocking peptide. The signal for CRY1 disappears across the retina when the peptide blocks the epitope targeting region. The signal from SWS1 remains unaffected. (C) Alexa Fluor 488 and Alexa Fluor 555 to the tissue without treatment with primary antibody (PBS instead). The absence of signal confirms that the signal comes only from secondary antibodies bound to their corresponding primary species. In the channel with Alexa 488, a faint background is visible across the entire section. The bright dots observable in every channel correspond to oil droplets in the cone photoreceptors. Bar in A and B is 50 µm and 10 µm in C.

# Tables

## Table S1. List of zebra finches, tissue collected and experimental conditions*.

| **Experiment** | **Bird ID** | **Sex** | **Retina collected** | **Fixation time** |
| --- | --- | --- | --- | --- |
| Full spectrum light control – AB signal detection in cryosection | 32 | Female | Right | 2h |
|  | 58 | Male | Left | 2h |
|  | 71 | Male | Right | 20 min |
| Full spectrum light control – AB signal detection in whole mount | 23 | Male | Right | 2h |
|  | 61 | Male | Right | 20 min |
|  | 62 | Female | Right | 20 min |
|  | 73 | Male | Left | 20 min |
| Full spectrum light control – AB signal quantification in whole mount | 55 | Female | Right | 2h |
|  | 56 | Male | Both | 2h |
|  | 57 | Male | Both | 2h |
| 463 nm light pre-exposure – AB signal detection in whole mount | 49 | Male | Both | 2h |
|  | 50 | Female | Both | 2h |
|  | 65 | Female | Left | 20 min |
| 521 nm light pre-exposure – AB signal detection in whole mount | 53 | Male | Both | 2h |
|  | 54 | Female | Both | 2h |
|  | 64 | Male | Right | 20 min |
| 638 nm light pre-exposure – AB signal detection in whole mount | 51 | Male | Both | 2h |
|  | 52 | Female | Both | 2h |
|  | 66 | Male | Right | 20 min |
| Full spectrum light control – AB signal detection & Peptide block control in cryosection | 60 | Male | Left | 20 min |
| Full spectrum light control - peptide blocking control in whole mount | 70 | Male | Left | 20 min |
| Full spectrum light control – Western Blot | 67 | Male | Both | 1h |
|  | 68 | Male | Both | 1h |

* In all the AB signal detection, quantifications and pre-exposure experiments, a “No primary antibody control” was included, producing no detectable signal (an example of such is shown in Fig S2A.).
